# A Critical Review of the Enhanced Recovery of Rare Earth Elements from Phosphogypsum

**DOI:** 10.3390/molecules28176284

**Published:** 2023-08-28

**Authors:** Gang Xie, Qingjun Guan, Fujia Zhou, Weijian Yu, Zhigang Yin, Honghu Tang, Zhenyue Zhang, Ru’an Chi

**Affiliations:** 1 China Nonferrous Metal Industry Technical Development and Exchange Center Co., Ltd., Beijing 100038, China; 15010016495@163.com; 2 Hunan Province Key Laboratory of Coal Resource Clean-Utilization and Mine Environment Protection, Hunan University of Science and Technology, Xiangtan 411201, China; ywjlah@163.com; 3 School of Resource Environment and Safety Engineering, Hunan University of Science and Technology, Xiangtan 411201, China; kunjia2012@foxmail.com; 4 Tianqi Lithium Corporation, Chengdu 610213, China; yinzg@tianqilithium.com; 5 Lithium Resources and Lithium Materials Key Laboratory of Sichuan Province, Tianqi Lithium Corporation, Chengdu 610000, China; 6 School of Minerals Processing and Bioengineering, Central South University, Changsha 410083, China; honghu.tang@csu.edu.cn; 7 School of XingFa Mining Engineering, Wuhan Institute of Technology, Wuhan 430073, China; 8 Hubei Three Gorges Laboratory, Yichang 443007, China; rac@wit.edu.cn

**Keywords:** rare earth elements, phosphogypsum, secondary resource, enhanced leaching, recrystallization, RIL

## Abstract

The increasing demand for rare earth elements (REEs), especially from new and innovative technology, has strained their supply, which makes the exploration of new REE sources necessary, for example, the recovery of REEs from phsophogypsum (PG). PG is a byproduct during the wet production of phosphoric acid, which is an attractive secondary resource for REEs due to a large amount of REEs locked in them. In most cases, REEs contained in PG are mainly encapsulated in the gypsum crystal, leading to a low leaching efficiency. Therefore, it is particularly important to use various methods to enhance the leaching of REEs from PG. In this review, we summarized and classified various enhanced leaching methods for the recovery of REEs from PG, and the advantages and disadvantages of different methods were compared. A joint method of recrystallization and RIL may be a promising enhanced leaching approach for the recovery of REEs from PG. Recrystallization could achieve both the complete REE release and simultaneous preparation of industrial materials with high value added, such as high-strength α-hemihydrate gypsum by phase transformation of PG, and the RIL technology could adsorb the releasing REEs and realize their efficient extraction. Such a combination appears to show significant advantages because of high REE recovery, as well as high value-added product preparation at low cost.

## 1. Introduction

Rare earth elements (REEs), also called rare earth metals or rare earths, are a set of 17 nearly indistinguishable metallic elements, which include the 15 lanthanides on the periodic table plus scandium and yttrium. REEs are necessary components of more than 200 products across a wide range of applications, especially high-tech consumer products and significant defense fields, such as cellular telephones, electric and hybrid vehicles, guidance systems, lasers, and radar and sonar systems [1].

**Figure 1:**
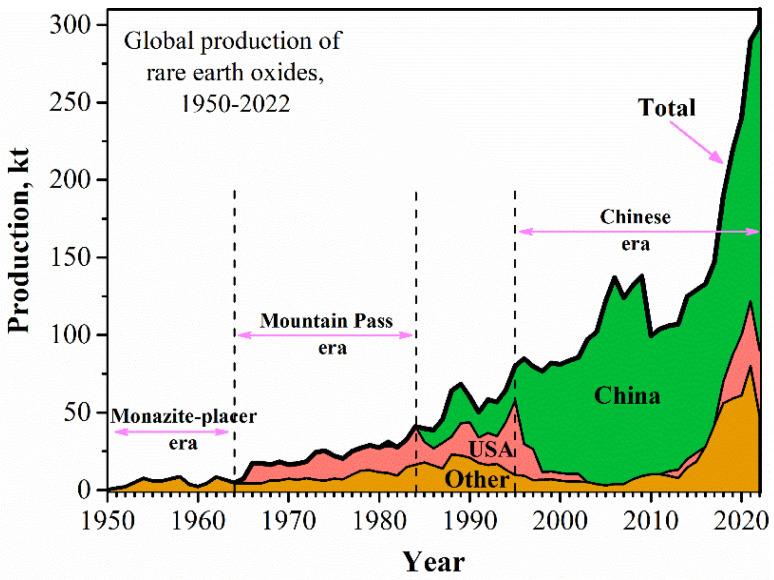
Global production of REEs (rare earth oxide equivalent) from 1950 to 2022, source: USGS.

**Figure 2:**
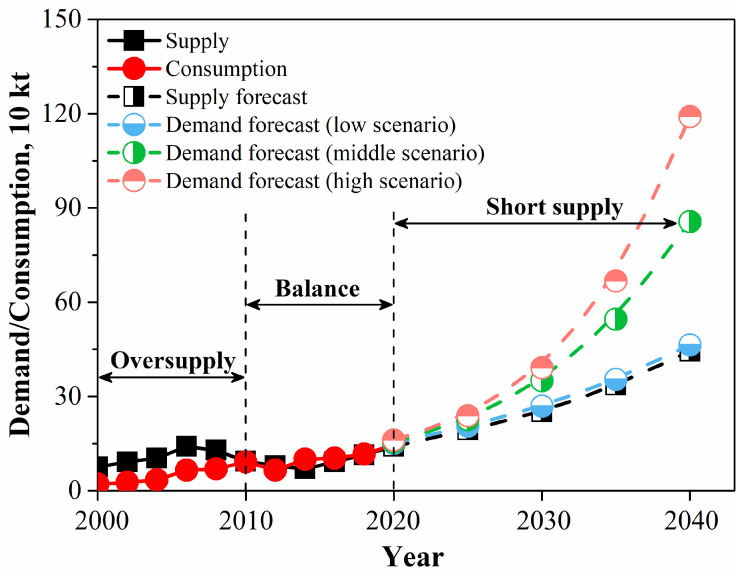
The consumption and demand of REEs (rare earth oxide equivalent) in China from 2000 to 2040 [8].

The global production of REEs (rare earth oxide (REO) equivalent) has gone through three main eras, including the Monazite-placer era, the Mountain Pass era, and the Chinese era from the 1950s until now (Figure 1). At present, REEs are predominantly produced from the minerals bastnasite, monazite, and loparite and the lattice ion-adsorption clays [2,3,4,5]. However, their increasing demand, especially from new and innovative technology, has strained supply (Figure 2) [6,7,8], which makes the exploration of new REE sources necessary, such as the recovery of REEs from end-of-life products and secondary sources [9,10,11]. For example, the US Department of Energy continues to fund research for cost-effective methods to extract REEs from coal and coal byproducts to develop substitutes for traditional REE resources and diversify their supply [12,13,14]. In addition, an enormous amount of research has focused on the extraction of REEs from PG.

PG is the main byproduct during the production of phosphoric acid through sulfuric acid digestion of phosphate rocks according to the following chemical reaction [15,16,17].

Ca_5_F(PO_4_)_3_ + *n*H_2_O + 5H_2_SO_4_ → 5CaSO_4_·*n*H_2_O + 3H_3_PO_4_ + HF

*where n is 0 for anhydrite (AH), 0.5 for bassanite (HH), and 2 for dihydrate gypsum (DH).*

Based on the crystalline water of gypsum, the wet production of phosphoric acid can be divided into the anhydrate process, hemihydrate process, dihydrate process, and their combinations. The classical dihydrate process is still the main process used to produce phosphoric acid in fertilizer industries, and the anhydrate process has not yet been industrialized [16]. Moreover, HH is in a metastable state, and it will slowly change into DH over time. Therefore, PG is mainly composed of DH. Generally, 4–5 tons of PG are generated for each ton of product acid (P_2_O_5_) [18]. So far, more than a billion tons of PG have been produced and mostly piled on land, leading to land occupation problems [19]. Moreover, the impurities contained in PG, such as radioactive elements of ^235^U, ^226^Ra, ^232^Th [20,21], and heavy metals of Cd and Ni [17], would be released into the surrounding environment and cause ecopollution over long-term storage [22]. Even worse, an estimated annual worldwide increase of 200–280 tons PG deteriorates the situation [23,24,25,26]. To utilize the large amount of PG, many studies have focused on transferring it into building materials, agricultural fertilizers, or soil stabilization amendments and as a set retarder in the manufacture of Portland cement in recent decades [27,28,29,30]. However, the applications of these PG products are severely limited by the contained impurities. From another point of view, impurities such as REEs might make PG a secondary resource. Phosphate rocks generally contain 0.046 wt% REEs on average, and more than 70% of them end up in the PG during sulfuric acid digestion [31]. Therefore, PG is considered a potential source of REEs [9,32,33]. More importantly, the removal of REEs and other hazardous elements from PG can greatly improve the value and utilization fields of PG products.

The REE content in PG varies greatly in different regions: some are less than 0.01 wt%, and some are as high as 6.8 wt%. Mineral acids and some organic acids are commonly used leaching agents during the recovery of REEs from PG in laboratory experiments. Among them, H_2_SO_4_ is considered an ideal agent because of its easy availability and low cost. However, in most cases, only low REE leaching efficiencies ranging from 12 to 40% have been obtained in sulfuric acid solution under normal lab conditions, as shown in Table 1. Lokshin et al. [34] attributed this to the inefficient diffusion of proton, sulfate ion, and rare earth ions between layers of sulfate groups that were tightly bound by calcium ions, and Walawakar et al. [26] suggested the low solubility of gypsum in sulfuric acid solutions caused by the common-ion effect should be responsible for the poor leaching efficiencies. Additionally, the formation of sparingly soluble double REE sulfates in H_2_SO_4_ solutions may also result in the difficult extraction of REEs from PG [33,35]. Although different researchers have different explanations for the poor leaching effect of REEs, most of them imply that the deposition form of REEs has a decisive effect on the leaching efficiency of REEs from PG [34]. Concerning the deposition form of REEs in PG, the possibilities are taht (i) REEs are structurally incorporated into the gypsum crystal lattice in the form of isomorphous substitution [36,37,38]; (ii) REEs precipitate as a separate phase such as oxides and sulfates trapped inside the gypsum crystal [39]; and (iii) REEs with phosphate and sulfate as counterbalancing ions occur as a metastable amorphous or nanocrystalline precipitate adsorbed onto the gypsum’s surface [40]. Compared to form (iii), forms (i) and (ii) of REEs are more difficult to leach from PG. The occurrence of REEs in PG can be roughly inferred from the leaching effect of H_2_SO_4_ under normal conditions: a low leaching efficiency indicates that REEs mainly exist in forms (i) and (ii) in the PG sample; on the contrary, a high leaching efficiency implies that REEs may exist in form (iii) in the PG sample. Consequently, REEs contained in PG are mainly trapped in the gypsum crystal lattice in most instances, which leads to the low leaching efficiency of REEs from PG in H_2_SO_4_ solutions.

In order to intensify the REE leaching from PG, many methods are carried out. However, there are few systematic summaries and comparisons of the various enhanced leaching methods. In this review, we concluded various intensified leaching methods and divided them into five categories: physically enhanced leaching methods, chemically enhanced leaching methods, phase inversion enhanced leaching methods, bioleaching methods, and joint methods. And the advantages and disadvantages of the different methods were compared.

Based on the leaching results, we concluded that the trace elements (e.g., U, Cd, and REE) are incorporated into the PG crystal lattice, which may explain their low concentrations in the leachates. Consequently, the total digestion of the PG matrix is required to solubilize REEs.

## 2. Physically Enhanced Leaching Methods

In order to intensify the REE leaching from PG in H_2_SO_4_ solutions, physically enhanced leaching methods, including mechanical activation, increasing the liquid/solid ratio or number of leaching, extending the leaching time, and ultrasonic or microwave treatment, are used to process PG.

Todorovsky et al. [56] explored the effect of mechanoactivation in different media (air, water suspension, and acid suspension) on the REE leaching from PG in a centrifugal ball mill. They found that activation in air caused a sharp decrease in the REE leaching in water. This was mainly ascribed to the partial dehydration of PG to form HH during the mechanoactivation in air, which led to the increased solubility of CaSO_4_ in water and to stronger bonding of the REEs to the hemihydrate. In addition, the activation caused a considerable increase in the specific surface and the crystal defects, which resulted in a high REE leaching efficiency of 70% in 7% H_2_SO_4_ solution with water as the activation media.

Liang et al. [47] obtained a maximum REE leaching efficiency of 43% from PG containing 218.42 ppm REEs in 5% H_2_SO_4_ solution under the condition of an increased contact time and S/L ratios. Lokshin et al. [34] aimed to increase the REE leaching efficiency by prolonging the leaching time. However, even if PG was leached for 18 weeks (3025 h), the REE leaching efficiency was only three times higher than that of leaching for 1 h in 0.5–4.0 wt% H_2_SO_4_ with an S/L ratio of 1/2.

**Figure 3:**
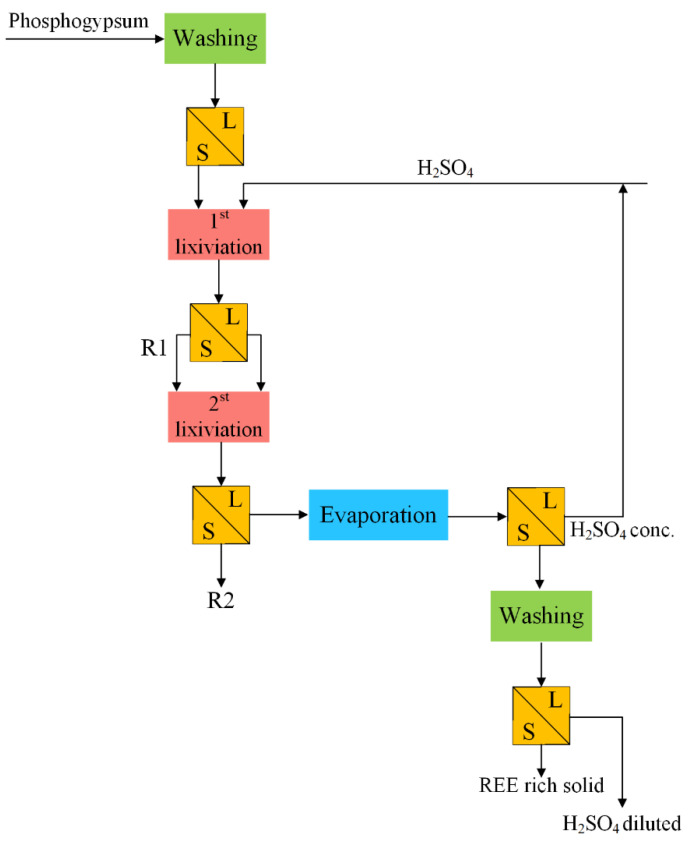
Flow diagram of the two-step H_2_SO_4_ leaching process [57].

Hammas-Nasri et al. [57] proposed a two-step H_2_SO_4_ leaching process to improve the REE recovery and purify PG, as shown in Figure 3. The method performed for concentrating REEs from PG (REE conc. of 224.93 ppm) consisted of the double leaching of the bulk sample with diluted sulfuric acid (10%) at 60 °C and evaporation of the liquor acid until the crystallization of a mixture of anhydrite-monetite phases rich with REEs (1671.89 ppm). The total enrichment of REEs in the crystallized solid during the process was approximately 86%. The analytical results showed that a double lixiviation was more efficient than a single one, as it allowed for better solubilization of REEs. However, the evaporation process was energy-consuming and inefficient when considering scaling up the process.

**Figure 4:**
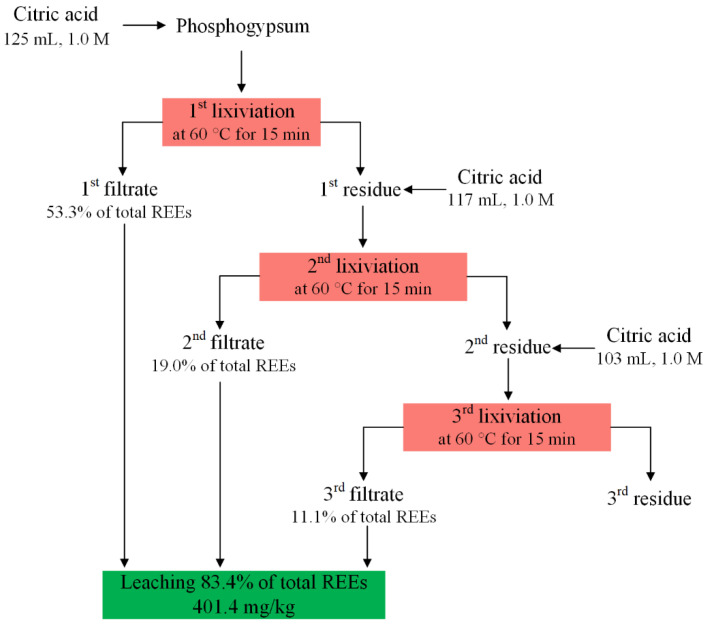
Three sequential leaching applications of REEs from PG using citric acid [55].

Gasser et al. [55] achieved a maximum leaching of 83.4% with three sequential leaching cycles for each leaching of 15 min using 1.0 mol/L citric acid solution at an L/S ratio of 5/1 and 60 °C. The detailed leaching procedure is shown in Figure 4.

**Figure 5:**
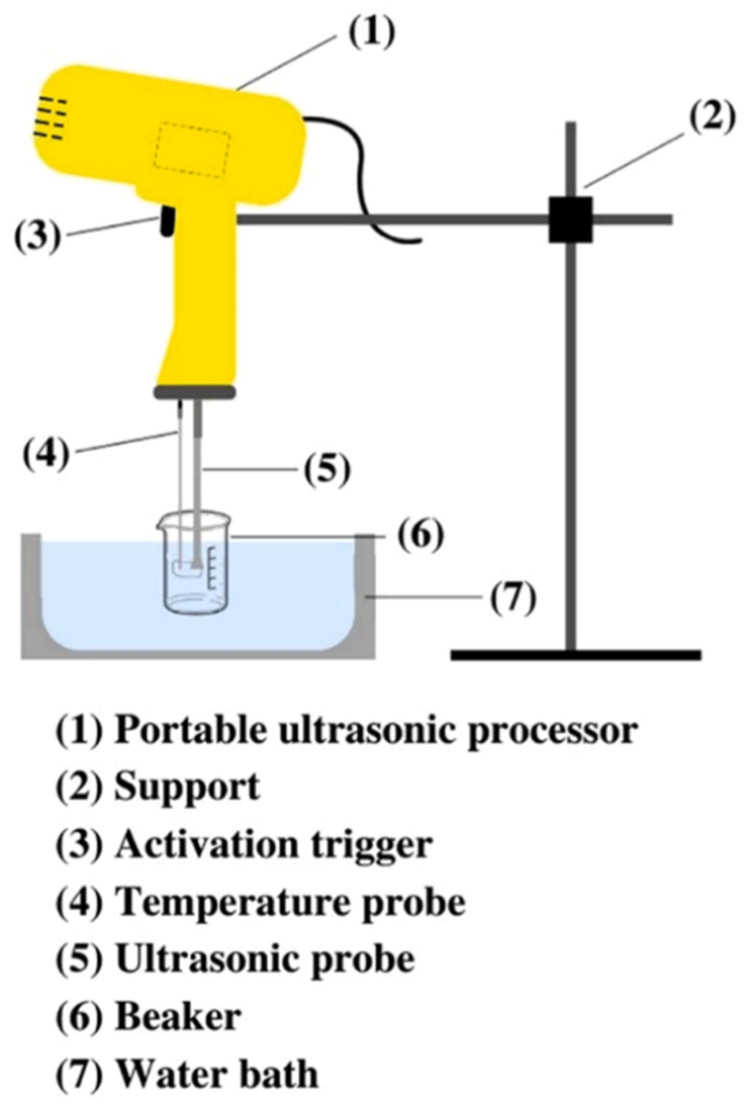
Experimental setup of the ultrasound-assisted leaching [51].

An ultrasonic treatment was also used to enhance the REE leaching from PG in H_2_SO_4_ solutions [58]. Lütke et al. [51] achieved a maximum leaching efficiency of 84% in 0.6 mol/L sulfuric acid solution using an ultrasonic amplitude of 77% and a pulse of 93.6% in the device shown in Figure 5, which was approximately 20% higher than that obtained in conventional leaching. In addition, the ultrasonic treatment could also significantly increase the leaching rate and shorten the leaching time. They ascribed the improvement in the REE leaching to the acoustic activation effects, which mainly led to a considerable reduction in the PG particle size.

The microwave process proved to be an effective approach to intensify the REE recovery from PG [59]. Lambert et al. [48] found that an optimal REE leaching was achieved by either microwaving at a low power (600 W) and short duration (5 min) or at a high power (1200 W) and long duration (15 min). The former creates cracks and pores in PG particles, enhancing the infiltration of the lixiviant. The latter results in the thermal degradation of PG particles and the release of REEs at the cost of the gypsum phase transition. In all cases, microwave pretreatment had a positive effect (more than 20% increase) on the REE leaching efficiency. At the optimum microwaving conditions, an 80% Nd, 99% Y, and 99% Dy leaching efficiency was achieved.

From the abovementioned discussion, although increasing the liquid/solid ratio or number of leaching applications with fresh leach solutions for each extraction could effectively promote the REE leaching from PG, this greatly reduced the REE concentration in the leachate and increased the amount of leachate, which increased the processing cost and made it difficult to industrialize. Increasing the number of leaching applications with the same leaching solution could effectively enrich the REEs in the leachate but had little effect on improving the leaching efficiency of REEs from PG. Moreover, the ultrasonic or microwave treatment was difficult to industrialize because of equipment limitations. Therefore, from the perspective of leaching efficiency and industrialization prospects, the mechanical activation seems to be an ideal method among all physically enhanced leaching methods.

## 3. Chemically Enhanced Leaching Methods

Chemically enhanced leaching methods mainly include the resin-in-leach (RIL) process and organic liquid leaching, which mainly intensified the REE recovery from PG through the chemical combination between additives and REEs.

### 3.1. RIL Technology

**Figure 6:**
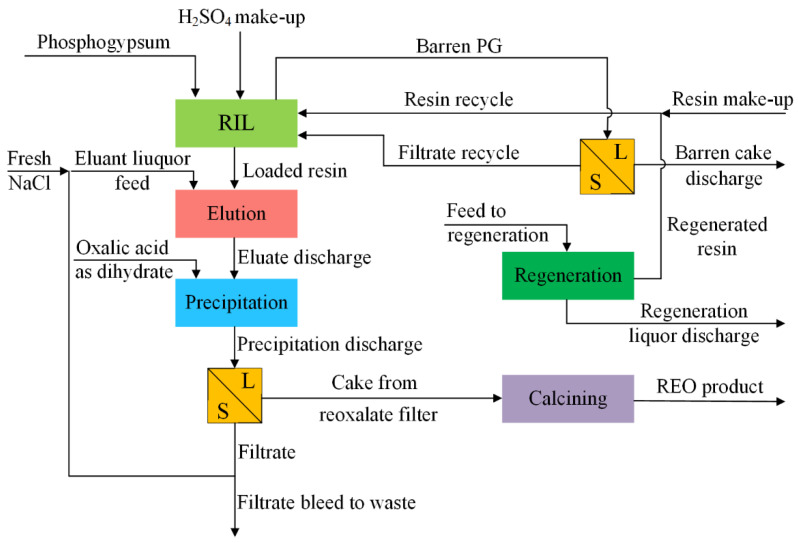
The flow diagram of the RIL technology for REE recovery from PG [61].

The addition of a sorbent into a PG suspension can adsorb REE ions from the solution, which results in an increase in the concentration gradient providing for the dissolution of sparingly soluble REE compounds and improving the REE leaching from PG. Based on this, researchers first introduced the resin-in-leach, RIL (alternatively: resin-in-pulp, RIP), process to the REE recovery from PG in the 1990s [60]. Usually, strongly acidic exchange cation resins containing a sulfonic acid group were used for REE recovery from PG suspensions [61]. Rychkov et al. [62] used a sulfonic cation exchange resin for the immediate separation of REEs from PG suspensions, which not only increased the REE recovery but decreased the sulfuric acid consumption. Kolyasnikov et al. [63] applied a new ion exchange resin containing both sulfonic and phosphonic exchangeable groups to separate calcium from REEs at the sorption stage and decreased the cost of eluates treatment. Yahorava et al. [61] optimized the RIL technology for REE recovery from PG in sulfuric acid media using a strong cation resin and presented the techno-economic analysis of the proposed flowsheet, as shown in Figure 6.

Although the strong cation resins containing a sulfonic acid group regenerated easily, their selectivity was poor. In order to solve the problem, the chelating resins, whose selectivity was far superior to the strong cation resins, were used in the RIL process [64]. However, they had the disadvantage of having an affinity towards other trivalent impurities present in solutions (Fe^3+^, Al^3+^, and Cr^3+^) and requiring strong eluents for regeneration. To look for environmentally benign eluents in the RIL process using the chelating resins, Santeri et al. [65] examined various eluent candidates and found MGDA and GLDA were both suitable candidates for effective REE elution from the chelating resins.

The benefits of the RIL process are the use of dilute H_2_SO_4_ as a lixiviant, a higher REE recovery through a leaching reaction driven in the forward direction and a simultaneous leaching and recovery step. At a price of USD > 21/kg for a mixed REE oxide product in conjunction with an overall REE recovery as low as 15%, the economics of REE recovery from PG via the RIL technology may already be favorable. However, this technology requires significant financial investment and its profitability is very sensitive to fluctuations in REE prices [61].

### 3.2. Solvometallurgical Method

The solvometallurgical method is referred to as organic extractant leaching in comparison to hydrometallurgy, where mineral acids are usually used. The method allows for a high selectivity of metal elements to be obtained and reduces both the consumption of acids and the volumes of leaching solutions [20,32].

**Figure 7:**
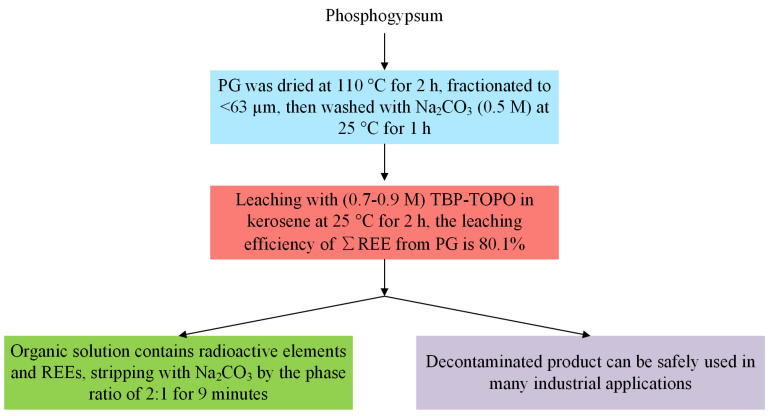
The flow diagram for REE recovery from PG using organic extractant leaching [67].

H. El-Didamony et al. [66,67] systematically investigated the leaching of radionuclides and REEs from PG using organic extractants. They found that kerosene was a more suitable diluent compared with toluene, benzene, and *n*-hexane, and tributyl phosphate (TBP) was a suitable organic extractant among the four types of organic extractants, including TBP, trioctyl phosphine oxide (TOPO), triphenyl phosphine oxide (TPPO), and di-ethyl-hexyl phosphoric acid (DEHPA). Under the optimum conditions, 69.8% of REEs from PG could be obtained using TBP as the leaching agent and kerosene as the diluent after two successive leaching steps. Using a mixture of TBP and TOPO with molar concentrations of 0.7 and 0.9, respectively, the leaching agent would further improve the REE leaching from PG. Further investigation showed that pretreating PG with hot sodium carbonate (0.5 M) prior to leaching with a mixture of TBP and TOPO in kerosene had a significant effect on the REE recovery, with an optimal leaching efficiency of 80.1%. The flow diagram is shown in Figure 7.

The organic extractant leaching demonstrated a high recovery of REEs, which should be the deposition form (iii) in PG. However, the method had difficulty in extracting REEs locked in the gypsum crystal. In addition, the loss of organic reagents that were generally adsorbed and entered into the matrix of gypsum may lead to high costs because of the amount of organic solvents used, as well as environmental concerns.

## 4. Phase Inversion Enhanced Leaching Methods

This method including carbonation and recrystallization mainly enhanced the leaching of REEs through the phase transition of PG. During the process, the locked REEs were released, migrated, and enriched into the leachate or a certain solid phase that could be easily attacked in the acid solution. Moreover, the newly formed solid material tended to be of higher value and wider applications, which would greatly reduce the cost of this process.

### 4.1. Carbonation

With the hydrothermal conversion of PG to an insoluble residue of calcium carbonate via treatment either with ammonium or sodium carbonate, as shown in Equations (1) and (2), researchers achieved preliminary enrichment of REEs in an easily leachable solid phase [68,69,70]. This technology was also considered an interesting method of up-concentrating REEs prior to their recovery through the dissolution of CaCO_3_ using mineral acids [71].

CaSO_4_·2H_2_O + Na_2_CO_3_ → CaCO_3_ ↓ + Na_2_SO_4_ + 2H_2_O (1)

CaSO_4_·2H_2_O + (NH_4_)_2_CO_3_ → CaCO_3_ ↓ + (NH_4_)_2_SO_4_ + 2H_2_O (2)

**Figure 8:**
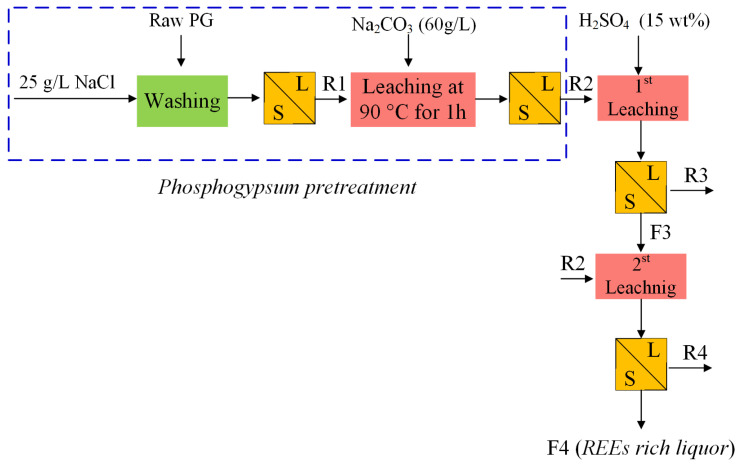
Method for REE recovery from PG using the hydrothermal conversion of PG to carbonate matrix [73].

**Figure 9:**
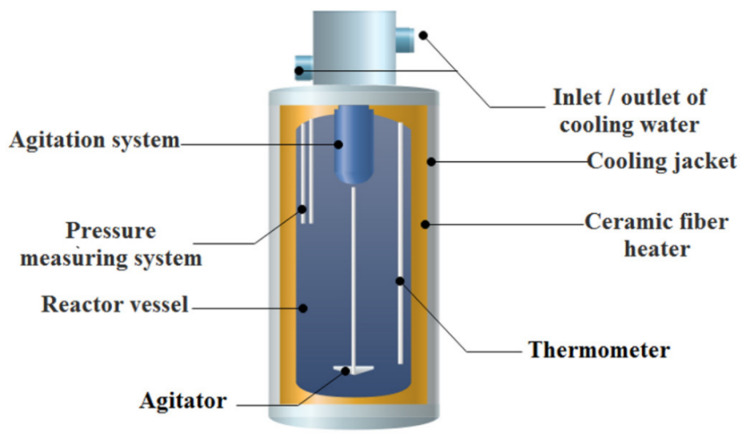
Descriptive scheme of a high-temperature/high-pressure reactor [73].

As shown in Figure 8, Masmoudi-Soussi et al. [72,73] converted PG into calcium carbonate according to Equation (1) with 60 g/L sodium carbonate solution at 90 °C for 1 h and enriched REEs into the newly formed solid phase. The total REE content increased sharply from 350.21 ppm in the original PG to 2250.31 ppm in the final residue. Rare earths’ migration to the carbonated matrix might be ascribed to the following two reasons: the first may be linked to the insertion of REEs into the gypsum matrix by replacement of calcium ions in PG, so while forming the calcium carbonate, which is more stable than the calcium sulfate, REEs migrate to the carbonated matrix by following the calcium ions [68]; the second may be related to the known affinity of REEs towards carbonates in an alkaline pH [74]. Then, using a sulfuric acid solution (15%) to separately leach two batches of the newly formed solid phase, at 100 °C for 2 h with a liquid/solid weight ratio of 3/1 in the setup shown in Figure 9, a final leach liquor with a total REE content of 4309 mg/L was obtained, as well as two anhydrite solids that could be used safely in industrial applications. Finally, the REE recovery from the sulfuric liquor was achieved by fractional precipitation with ammonia (10%).

**Figure 10:**
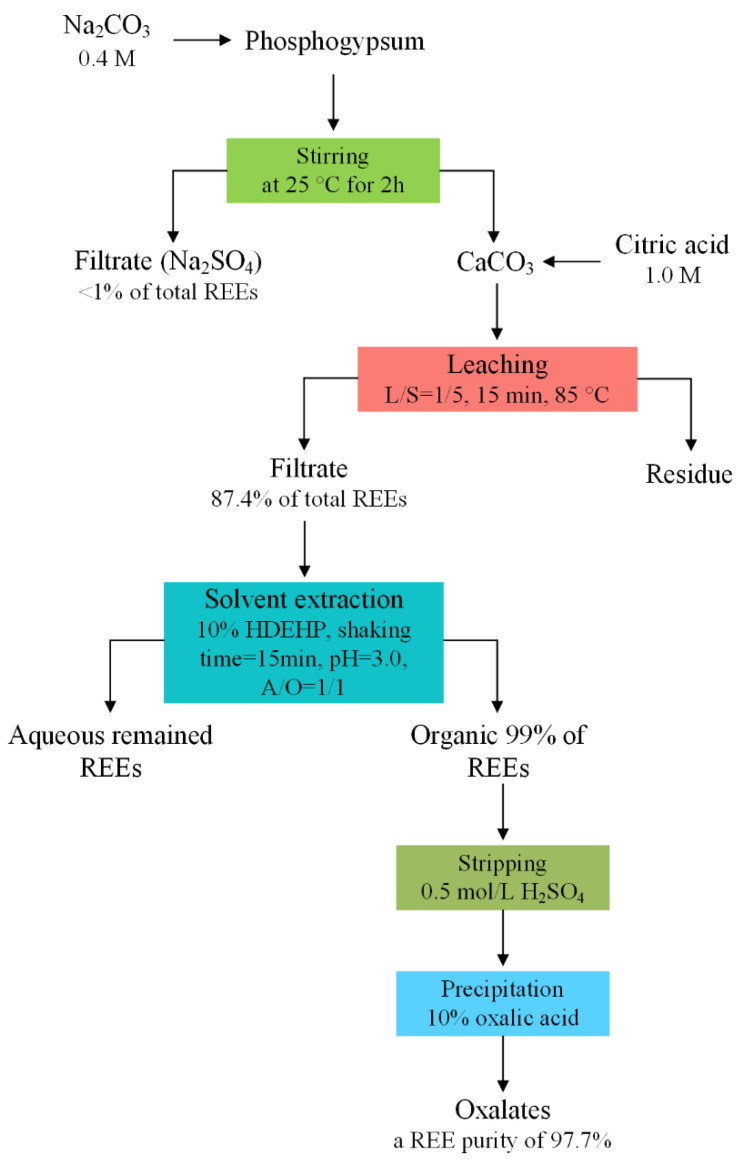
Flow diagram for REE leaching from PG with carbonate transformation followed by citric acid leaching [69].

Gasser et al. [69] also enriched REEs into the carbonate matrix that is easily attacked in acid solution by transforming calcium sulfate in PG to calcium carbonate through reacting with sodium carbonate at 25 °C, and then REEs precipitated with calcium carbonate were leached out by the use of citric acid with a maximum leaching of 87.4% and further purified through solvent extraction and precipitation. The process is shown in Figure 10.

The major disadvantage of the carbonation process was a high reagent cost and energy consumption, as well as the limited market for CaCO_3_ and (NH_4_)_2_SO_4_ as byproducts [61].

### 4.2. Recrystallization

The mechanism of α-HH or AH formation using the hydrothermal method is a reaction of DH dissolution in solutions, followed by crystallization (the so-called through-solution reaction mechanism) [75,76]. During the DH dissolution, the impurities encapsulated in gypsum crystals can be completely liberated into the solutions. Based on the mechanism, Liu et al. [77,78] achieved the sufficient removal of iron (removal efficiency > 99%) and chromium (removal efficiency > 99.5%) from byproduct gypsum by the conversion of DH into AH. Their work revealed that locked metal ions would be fully released during the dissolution process, and the addition of a mineralizer (such as HCl) could effectively adjust the occurrence mode of the released metal ions in solutions and prevent them from recombining with AH during the crystallization process, thus efficiently leaching metal impurities from byproduct gypsum. However, the process that involved heating at elevated temperatures (>120 °C) and pressures for more than 4 h was energy intensive.

**Figure 11:**
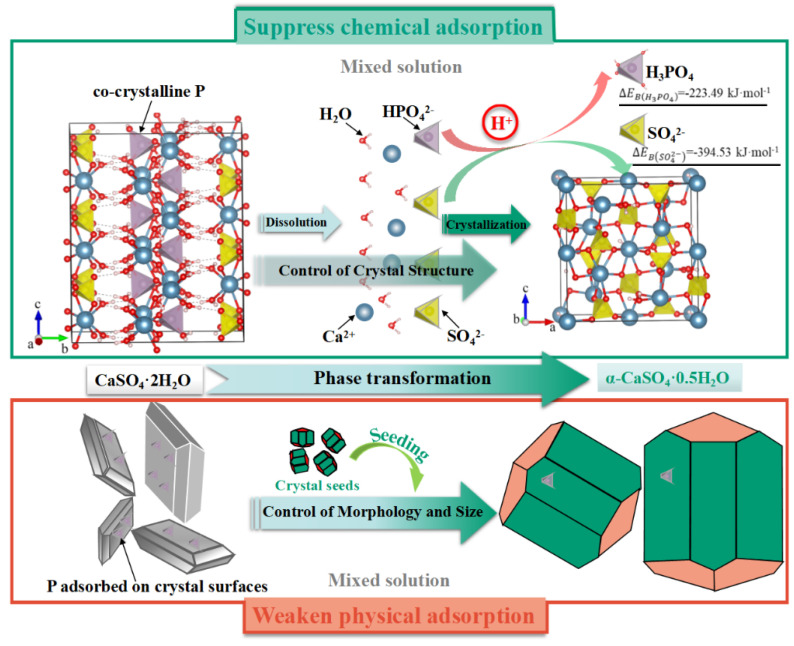
Mechanism of deep phosphorus removal from PG based on crystal regulation induced by seeding [83].

More recently, Guan et al. [18,79] obtained the deep removal of co-crystalline phosphorus (P) impurities and synchronous preparation of high-strength α-HH from PG through phase transition in a mixed solution of inorganic salt and mineral acid under mild conditions. The main role of inorganic salt was to facilitate the phase transition, during which the co-crystalline P was liberated entirely into the solutions. The main function of mineral acid was to regulate the species of the released P in the solution and, thus, inhibit their chemical recombination with calcium sulfate during α-HH crystallization. Moreover, α-HH’s shape and size were controlled via seeding. The formation of the high-strength α-HH, which were large, short-columnar crystals with low specific surface areas [80,81], suppressed the physical adsorption of P on the product’s surface and further increased the leaching efficiency to above 97%. They developed a combined process in which both the synthesis of high value-added products and P impurity extraction from PG could be instantaneously achieved. A diagram of the mechanism is shown in Figure 11. Furthermore, they also explored the migration and distribution of REEs in PG during the phase transformation from DH to α-HH in 2.0 M sulfuric acid solutions at 95 °C and an S/L ratio of 1/30. They found that although the dissolution process could fully release the REEs trapped in the gypsum lattice and lead to a rapid increase in the REE leaching efficiency, the fine crystals with high specific surface areas formed during the subsequent recrystallization process would adsorb the majority of dissolved REEs and eventually result in a sharp drop in the REE leaching efficiency. Accordingly, the crystal regulation of the recrystallized gypsum products was important for improving the REE leaching efficiency during the phase transformation. The addition of EDTA-2Na could control the crystal morphology and particle size of α-HH and promote the formation of thick and short columnar crystals with a small specific surface area, which greatly weakened the adsorption of dissolved REEs on the recrystallized gypsum crystal surfaces, thereby improving the REE leaching efficiency significantly from 33.0% to 59.3% after leaching for 2 h [31]. Moreover, the thick and short columnar α-HH was identified as high-strength gypsum with high value added [82].

The crystallization method involving efficient REE extraction and simultaneous preparation of recrystallized gypsum products with high value-added might be a low-cost, sustainable, and green solution to the PG problem. However, to achieve efficient extraction of REEs from PG through recrystallization, two requirements must be met: one is controlling the morphology and size of recrystallized crystals and forming regular large-grained crystals to reduce the surface adsorption; the other is regulating the species of REEs in solutions to avoid their re-entry into the recrystallized crystals. Therefore, the crystallization method is promising but requires more research and development before it can be performed on a large scale.

## 5. Bioleaching Methods

**Figure 12:**
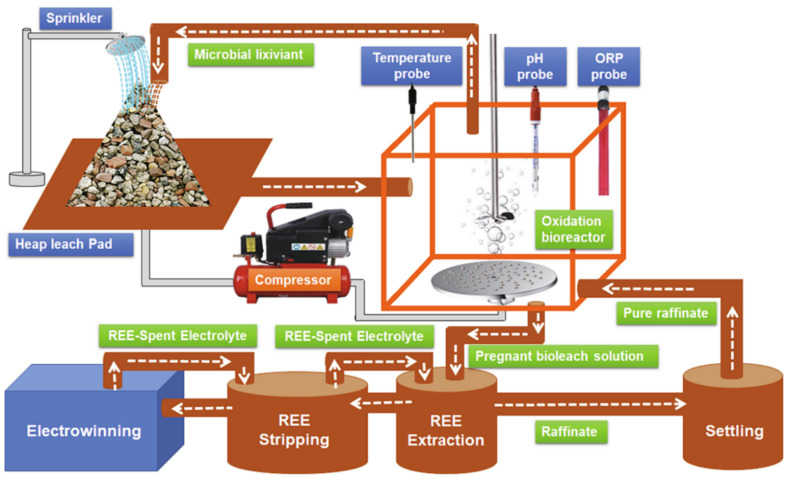
Schematic diagram of the field unit operation of the heap-bioleaching system [84].

The bioleaching technology for metal extraction can achieve a higher metal specificity and leaching efficiency at lower concentrations [84]. Moreover, this emerging technology can effectively avoid high operational costs and heavy metal pollution and sludge that occur when some chemical or physical–chemical strategies are used in the metal recovery [85,86]. Therefore, bioleaching technology is a very promising alternative for the recovery of REEs from waste materials [87,88,89]. Bioleaching is performed by both autotrophic and heterotrophic microorganisms, and the selection of the microorganism depends on the type of mineral. To extract REEs from PG by bioleaching, various microbial species, such as *Desulfivibrio*, *Acidithiobacillus*, and *Acetobacter*, can be used through heap and column leaching processes, as shown in Figure 12 [84]. Antonick et al. [50] explored the ability of a gluconic-acid-dominated biolixiviant produced by *Gluconobacter oxydans* NRRL B85 for the extraction of REEs from synthetic PG. They found that biolixiviant was more effective for REE leaching compared to a commercial gluconic acid, which implies that microbial effects could enhance the REE leaching. Studies found that a mixed culture of sulfur-oxidizing bacteria could leach 55–70% of REEs from PG within a 30-day incubation period at pH 1.5–1.8, which was mainly due to the sulfuric acid generated by these sulfur-oxidizing bacteria [90]. In addition, Tayar et al. [91] demonstrated that the mesophilic *Acidithiobacillus thiooxidans* possessed higher sulfuric acid production even compared to strains containing thermophilic archeas, which achieved higher REE extraction (98% Nd, 60% Ce, 58% La, and 62% Y) in the two-step bioleaching.

Although bioleaching has many advantages, it suffers from lower yield and rates. So far, only a little information is available for bioleaching REEs from PG. Consequently, there is still a long way to go for the industrial application of this method.

## 6. Joint Methods

The joint method uses more than one enhanced leaching method in the REE leaching from PG to achieve a higher REE leaching efficiency.

**Figure 13:**
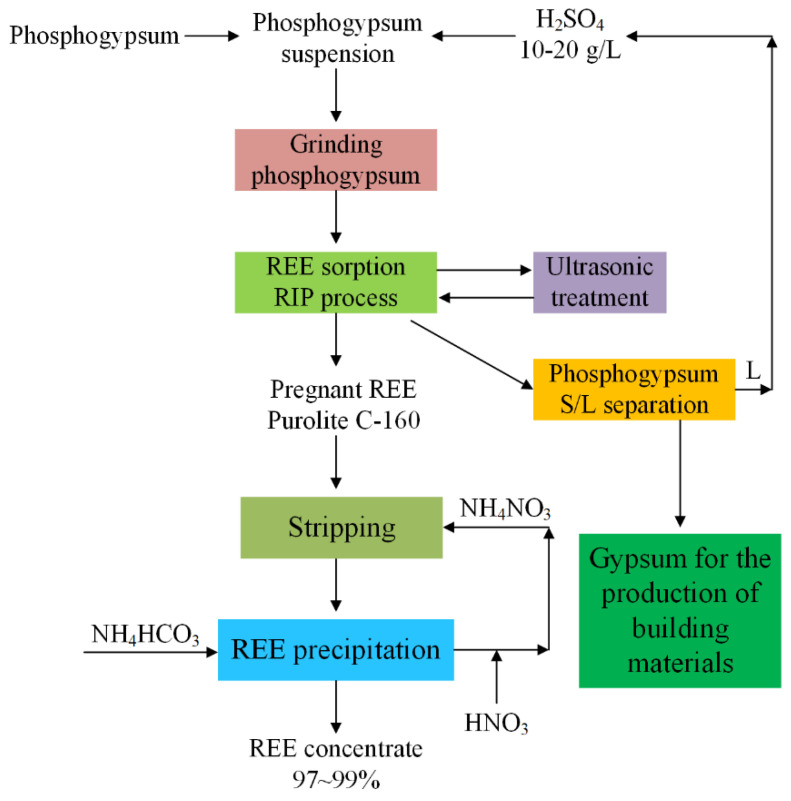
Flow diagram of the RIL technology combined with mechanical grinding and ultrasonic treatment for REE recovery from PG [35].

Rychkov et al. [35] systematically compared the effects of mechanical grinding, ultrasonic treatment, and the RIL process on the recovery of REEs from PG and found that mechanical grinding had the best activation effect on REE leaching, followed by the RIL process, and ultrasonic treatment had the worst activation effect. The simultaneous use of all three methods led to a significant increase in the leaching efficiency of REEs from PG from 15–17% to more than 70% in only 10–20 g/L sulfuric acid solutions. A concentrate containing approximately 50% of REEs (corresponding to 97–99% Ln_2_O_3_) was obtained using 400 g/L ammonium nitrate solution as an eluent followed by REE carbonate precipitation from the eluent by ammonium hydrogen carbonate solution. The process is shown in Figure 13.

**Figure 14:**
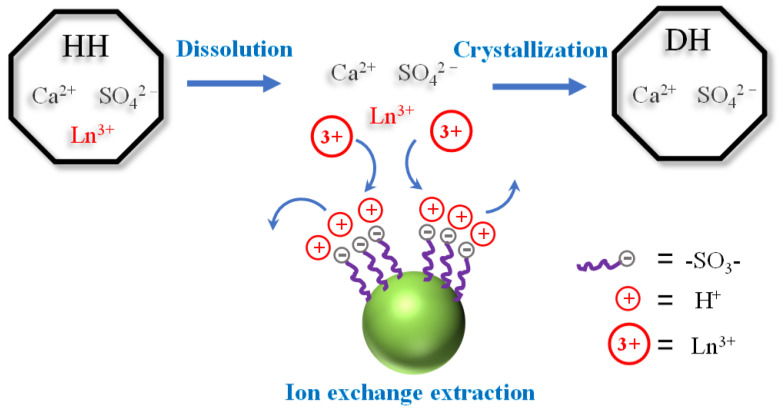
A schematic representation of the recrystallization of HH to DH (gypsum) with simultaneous removal of REEs by means of ion exchange [92].

In the hemidihydrate (HDH) phosphoric acid production process, the initially formed HH would be recrystallized into DH, which is a dissolution–crystallization process. And during the HH dissolution process, REEs trapped in the gypsum crystals would be released completely from HH. Koopman et al. [92] took advantage of the REE release effect during the HH dissolution process and added ion exchange resins during the phase transformation from HH to DH to achieve efficient recovery of REEs from PG. A schematic diagram of the process is shown in Figure 14.

Combining both recrystallization and RIL may be a promising approach for the recovering of REEs from PG. The recrystallization of PG to high-strength α-HH through the dissolution–crystallization process under mild conditions can fully release REEs trapped in the gypsum crystals, during which the addition of ion exchange resins can effectively adsorb the released REEs and achieve their efficient recovery. More importantly, the recrystallized product with high value-added has a wide range of applications and markets. Therefore, this joint method has the advantage of lower cost owing to the efficient extraction of REEs and synchronous preparation of high value-added products, which is conducive to its industrial application.

## 7. Conclusions

REEs plays a critical role in national security, energy independence, environmental future, and economic growth. Owing to the critical role, interest and research into the recovery of REEs from end-of-life products and secondary sources such as PG has recently increased. PG which contains a large amount of REEs is a bulk solid waste generated from wet-process phosphoric acid production. Because of the encapsulated state of REEs contained in PG, enhanced leaching seems to be necessary for the efficient extraction of REEs from PG. This review highlighted various enhanced leaching methods for the recovery of REEs from PG and compared the advantages and disadvantages of various methods, as shown in Table 2. Based on this, we suggest that a joint method of recrystallization and RIL may be a promising enhanced leaching approach for the recovery of REEs from PG. The recrystallization could achieve both the complete REE release and simultaneous preparation of industrial materials with high value-added, such as high-strength α-HH with the phase transformation of PG, and the RIL technology could adsorb the released REEs and realize their efficient extraction. Such a combination appears to show significant advantages because of the high REE recovery, as well as the high value-added product preparation at low cost.

## Figures and Tables

**Table 1:** Part of previous studies on REE leaching from PG.

Origin	Country	REO or REE (wt%)	Leaching Conditions					Leaching Efficiency, %	Ref.
			Leaching Regent	Conc. (M ^a^ or wt% ^b^)	Temp. (°C)	Time (h)	L/S (mL/g)		
Kola	Russia	0.6	H_2_SO_4_	10–15 ^b^	40	6	2/1	52	Jarosiński et al. [41]
Phosphoric Acid Plant at Phalaborwa	South Africa	6.8	HNO_3_	2.0 ^a^	20	48	3/1	57	Preston et al. [42]
Abu-Zaabal Company	Egypt	0.022	HNO_3_; Ca(NO_3_)_2_	2.0–3.0 ^a^; 0.8 ^a^	25	8	1/1	76	El-Reefy et al. [43]
			HNO_3_	2.0 ^a^				46	
			HCl	4.0 ^a^				30	
			H_2_SO_4_	4.0 ^a^				30	
Private Joint Stock Company ‘Metakhim’	Russia	0.414	H_2_SO_4_	0.5–4.0 ^b^	25	3025	2/1	57.1–68.2	Lokshin et al. [34]
Dump PG	Russia	0.45	H_2_SO_4_; HNO_3_	1.0–3.0 ^b^	—	8–12 min	4/1–5/1	85–86.1	Abramov et al. [44]
Synthetic PG	USA	0.034	H_2_SO_4_; H_3_PO_4_	25 ^b^; 96 ^b^	72	1	20/3	49	Al-Thyabat and Zhang [45]
Abu-Zaabal Company	Egypt	0.048	HNO_3_	3.0 ^a^	25	3	2/1	43.3	Ismail et al. [46]
			HCl	2.0 ^a^				11.9	
			H_2_SO_4_	4.0 ^a^				12.5	
Agrium Fertilizer Plant	Canada	0.020	HNO_3_	1.5 ^a^	80	2	8/1	57	Walawalkar et al. [26]
			HCl					51	
			H_2_SO_4_					23	
Mosaic Company	USA	0.0218	H_2_SO_4_	5.0 ^a^	50	3.5	4/1	43	Liang et al. [47]
Nutrien Ltd.’s Fertilizer Operations	Canada	0.0317	HCl	1.5 ^a^	85	1	15/1	80–99	Lambert et al. [48]
Huelva PG Stack	Spain	0.0345	H_2_SO_4_	0.5 ^a^	25	2–8	20/1	41–58	Cánovas et al. [49]
			HNO_3_	3.0 ^a^				75–86	
Synthetic PG	USA	1.0	H_2_SO_4_	0.22 ^a^	25	24	50/1	76.9–93.7	Antonick et al. [50]
			H_3_PO_4_					5–85	
Catarinense Carbochemical Industry S/A	Brazil	0.5	H_2_SO_4_	0.6 ^a^	42	1.0	20/1.7	67.8	Lütke et al. [51]
Catarinense Carbochemical Industry S/A	Brazil	0.5	H_2_SO_4_	2.9 ^a^	55	20 min	20/1.7	90	Lütke et al. [52]
Catarinense Carbochemical Industry S/A	Brazil	0.5	Citric acid	3.0 ^a^	80	1.0	20/1	62.0	Lütke et al. [52]
Yunnan Phosphate Chemical Group	China	0.02	HCl	1.65 ^a^	25	2.0	10/1	52	Guan et al. [53]
					60			66	
					80			78	
Yunnan Phosphate Chemical Group	China	0.02	HNO_3_	1.65 ^a^	30	2.0	10/1	58.5	Zeng et al. [54]
					60			75.9	
					80			83.4	
Abu-Zaabal Company	Egypt	0.048	Boric acid	0.5 ^a^	25	20	5/1	17	Gasser et al. [55]
			Malic acid	1.0 ^a^	25	15 min	5/1	17.7	
			Citric acid	1.0 ^a^	60	15 min	5/1	53.3	

**Notes: a** represents molar concentration (mol/L); **b** represents mass concentration (wt%).

**Table 2:** Overview of the advantages and disadvantages of the intensified leaching methods discussed in this review.

Methods		Advantages	Disadvantages
**Physically enhanced leaching methods**	Mechanical activation	● Simple and efficient	● High energy consumption
	Increasing L/S ratio or number of leaching applications	● Easy operation	● High cost of leaching solution treatment
	Extending the leaching time	● Easy operation	● Low efficiency
	Ultrasonic/microwave treatment	● Greatly promotes REE leaching ● Shortens the leaching time	● High energy consumption ● Difficult to achieve mass production
**Chemically enhanced leaching methods**	RIL technology	● Dilutes H_2_SO_4_ as a lixiviant ● A high REE recovery ● A simultaneous leaching and recovery step	● Significant financial investment ● Vulnerable profitability
	Solvometallurgical method	● A high recovery of REEs	● Difficult to extract REEs locked in the gypsum crystal ● Loss of organic reagents
**Phase inversion enhanced leaching methods**	Carbonation	● Enrichment of REEs in an easily leachable solid phase	● High reagent cost and energy consumption ● Limited market for byproducts
	Recrystallization	● Efficient REE extraction and simultaneous preparation of high-value-added gypsum products ● Low-cost, sustainable, and green solution	● Needs more research and development
**Bioleaching methods**		● High metal specificity and leaching efficiency at low concentrations ● Environmentally friendly ● Low operating cost	● Low yield and rates ● Few studies on the bioleaching of REEs from PG
**Joint methods**		● Efficient REE recovery ● Low cost ● Easy to industrialize	● Needs more research and development

## Data Availability

Not applicable.

## Sample Availability

Not available.
